# Odor, Not Performance, Dictates *Bemisia tabaci*'s Selection between Healthy and Virus Infected Plants

**DOI:** 10.3389/fphys.2017.00146

**Published:** 2017-03-16

**Authors:** Gong Chen, Qi Su, Xiaobin Shi, Xin Liu, Zhengke Peng, Huixin Zheng, Wen Xie, Baoyun Xu, Shaoli Wang, Qingjun Wu, Xuguo Zhou, Youjun Zhang

**Affiliations:** ^1^College of Plant Protection, Hunan Agricultural UniversityChangsha, China; ^2^Department of Plant Protection, Institute of Vegetables and Flowers, Chinese Academy of Agricultural SciencesBeijing, China; ^3^Institute of Insect Sciences, College of Agriculture, Yangtze UniversityJingzhou, China; ^4^Department of Entomology, University of KentuckyLexington, KY, USA

**Keywords:** mother-knows-best, plant virus, volatiles, performance, host preference, non-vector insects

## Abstract

Although, insect herbivores are generally thought to select hosts that favor the fitness of their progeny, this “mother-knows-best” hypothesis may be challenged by the presence of a plant virus. Our previous study showed that the whitefly, *Bemisia tabaci*, the obligate vector for transmitting *Tomato yellow leaf curl virus* (TYLCV), preferred to settle and oviposit on TYLCV-infected rather than healthy host plant, *Datura stramonium*. The performances of *B. tabaci* larvae and adults were indeed improved on virus-infected *D. stramonium*, which is consistent with “mother-knows-best” hypothesis. In this study, *B. tabaci* Q displayed the same preference to settle and oviposit on *Tomato spotted wilt virus* (TSWV)-infected host plants, *D. stramonium* and *Capsicum annuum*, respectively. As a non-vector of TSWV, however, insect performance was impaired since adult body size, longevity, survival, and fecundity were reduced in TSWV infected *D. stramonium*. This appears to be an odor-mediated behavior, as plant volatile profiles are modified by viral infection. Infected plants have reduced quantities of o-xylene and α-pinene, and increased levels of phenol and 2-ethyl-1-hexanol in their headspace. Subsequent behavior experiments showed that o-xylene and α-pinene are repellant, while phenol and 2-ethyl-1-hexanol are attractive. This indicates that the preference of *B. tabaci* for virus-infected plants is modulated by the dynamic changes in the volatile profiles rather than the subsequent performances on virus-infected plants.

## Introduction

Although, resource allocation by heterotrophs has been studied extensively, the extent to which their behavioral responses is manipulated by pathogens/parasites reflects the fine-tuned evolutionary adaptation in nature (Roy and Raguso, 1997; Ebbert and Nault, 2001; Eigenbrode et al., 2002; Hurd, 2003; Maris et al., 2004; Belliure et al., 2005; Lacroix et al., 2005; Lefèvre et al., 2006; Mauck et al., 2010; Bosque-Pérez and Eigenbrode, 2011). The choice of plant hosts for oviposition by insects remains an important research area in entomology and ecology (Bernays and Chapman, 1994; Schoonhoven et al., 2005; Tilmon, 2008). For insects, the preference-performance hypothesis (PPH) suggests that females will maximize their fitness by laying their eggs on plant types on which their offspring will perform best (Gripenberg et al., 2010). This hypothesis, also known as the optimal, oviposition theory (Jaenike, 1978), the naive adaptionist theory (Courtney and Kibota, 1990), or the “mother knows best” principle (Valladares and Lawton, 1991), was originally proposed by Jaenike (1978). Some studies testing female oviposition preferences and larval performance have established positive correlations between females and their offsprings (Craig et al., 1989; Heisswolf et al., 2005; Staley et al., 2009). Other studies, however, showed that mothers could make poor oviposition decisions, leading to a weak preference–performance relationship between female adults and offsprings (Rausher, 1979; Scheirs et al., 2004; Digweed, 2006; Gripenberg et al., 2007). These “bad motherhood” cases have caused extensive debates (Mayhew, 2001; Gripenberg et al., 2010). Alternative hypotheses include optimal foraging (Scheirs et al., 2000), enemy-free space (Denno et al., 1990), and insect neural capacity (Bernays, 2001).

The current study concerns the effects of virus infection of host plants on herbivorous insects. The influences of virus-infected host plants on herbivorous insects can be neutral (Matsuura and Hoshino, 2009), beneficial (Maris et al., 2004; Belliure et al., 2005), or deleterious (Rubinstein and Czosnek, 1997) depending on the specific combination of herbivore, virus, and plant. In addition, viruses can affect plant-derived volatiles such that herbivorous insects are repelled (van Molken et al., 2012) or attracted (Srinivasan et al., 2006; Ngumbi et al., 2007). These manipulations can strongly influence rates of virus transmission and thus have important implications for ecology, human health, and agriculture (Hurd, 2003; Lefèvre et al., 2006, 2009). Although, host choice by insects has been continually studied, the influences of virus infection of host plants on host choice by vector and non-vector insects are not well-understood.

*Datura stramonium* L. (Solanaceae), one of the hosts included in the current study, is a summer weed that originated in the Americas and that has a worldwide distribution (Holm et al., 1997). *D. stramonium* is also a host for many plant viruses, including *Tomato yellow leaf curl virus* (TYLCV) (Cohen and Nitzany, 1966) and *Tomato spotted wilt virus* (TSWV) (Moyer, 1999). TSWV (genus *Tospovirus*, family Bunyaviridae) infects and causes disease in hundreds of plant species, including many ornamentals and vegetables (Moyer, 1999). TSWV is transmitted in a propagative and circulative manner by thrips (*Thysanoptera*: *Thripidae*; Ullman et al., 1992).

The sweetpotato whitefly, *Bemesia tabaci* (Gennadius) (Hemiptera: Aleyrodidae), is a vector of Begomoviruses but not for TSWV. Although, there has been much debate about whether *B. tabaci* is “a species complex or complex of species” (Perring, 2001; Brown, 2010; Dinsdale et al., 2010; De Barro et al., 2011), the most current molecular evidence reveals that *B. tabaci* actually includes at least 24 genetically distinct but morphologically indistinguishable cryptic species (De Barro et al., 2011). The most damaging and widespread cryptic species are the Mediterranean genetic group (biotype Q) and the East-Minor Asia 1 genetic group (biotype B). In a field survey conducted in 2012, we observed *B. tabaci* Q outbreaks on TSWV-infected plants in several provinces of China, although *B. tabaci* Q is not a vector of TSWV. Previous study has demonstrated that TSWV infection can decrease *B. tabaci* Q performance on pepper (Pan et al., 2013). TYLCV infection, in contrast, increases *B. tabaci* Q (vector) attraction and performance on its host, *D. stramonium* (Chen et al., 2013).

Previous research examining virus effects on plant-insect interactions has focused primarily on vectors, whereas the effects of virus-infected plants on non-vectors have been largely overlooked (Su et al., 2016). In this study, we investigated the effects of TSWV infection on a non-vector *B. tabaci* Q. The outbreaks of *B. tabaci* Q were occurred on TSWV-infected pepper even though the performance of this non-vector was compromised. In addition, we test the volatiles released by TYLCV- and TSWV-infested *D. stramonium*. We hypothesized that both viruses induce similar changes in volatiles emitted by *D. stramonium* to make *B. tabaci* Q select virus-infested *D. stramonium*.

## Results

### *B. tabaci* Q settling and oviposition preferences on TSWV-infected vs. healthy *D. stramonium*

**Figure 1:**
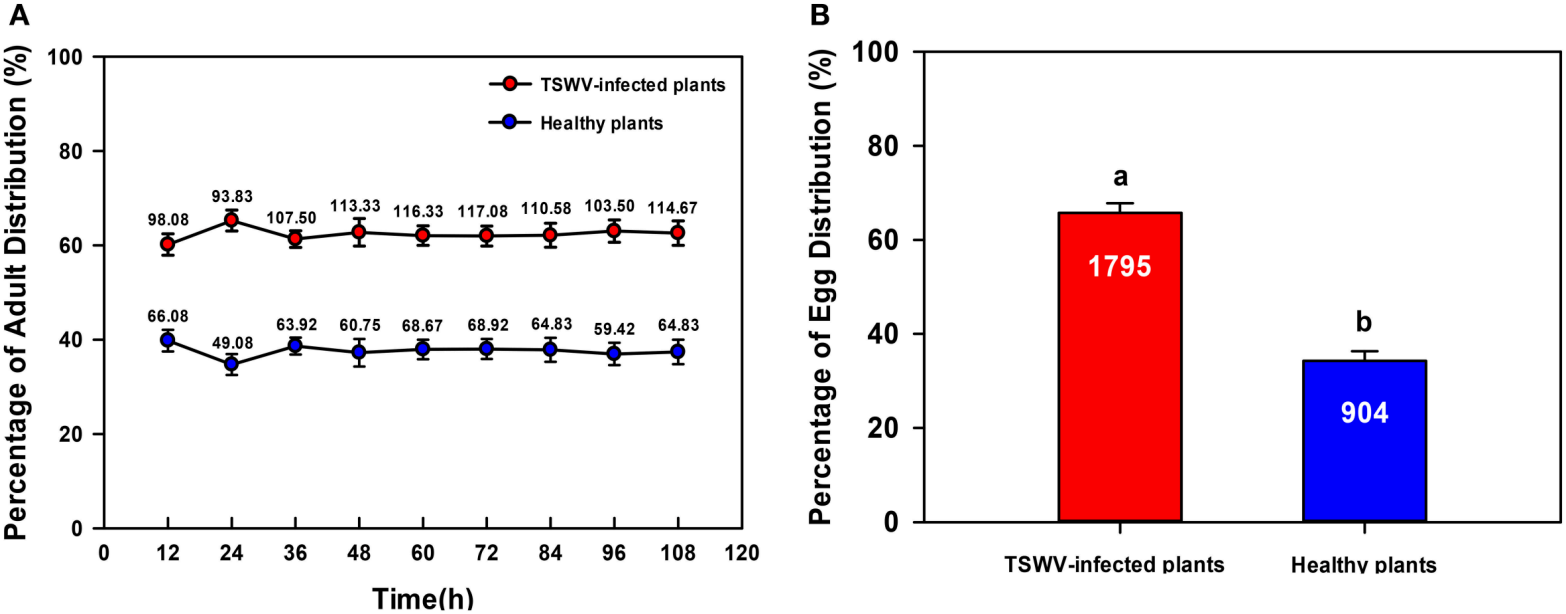
**Settling and oviposition of *B. tabaci* Q on healthy vs. TSWV-infected *D*. *stramonium***. **(A)** Percentage of adults that settled on healthy vs. TSWV-infected plants (*n* = 300, 12 replicates). **(B)** Percentage of eggs distributed on healthy vs. TSWV-infected plants. Values are means ± SE (One-way ANOVA). The number of eggs was shown inside the bar. Settling and oviposition were significantly greater on the TSWV-infected plants than on the healthy plants (*P* < 0.05).

Significantly more *B. tabaci* Q adults settled on TSWV-infected than on healthy *D. stramonium* [One-way ANOVA, *F*_(1, 22)_ = 91.147, *P* < 0.001; Figure 1A]. Similarly, *B. tabaci* Q adults deposited significantly more eggs on TSWV-infected than on healthy *D. stramonium* [One-way ANOVA, *F*_(1, 22)_ = 114.406, *P* < 0.001; Figure 1B].

### Life history traits of *B. tabaci* Q on TSWV-infected and healthy *D. stramonium*

**Figure 2:**
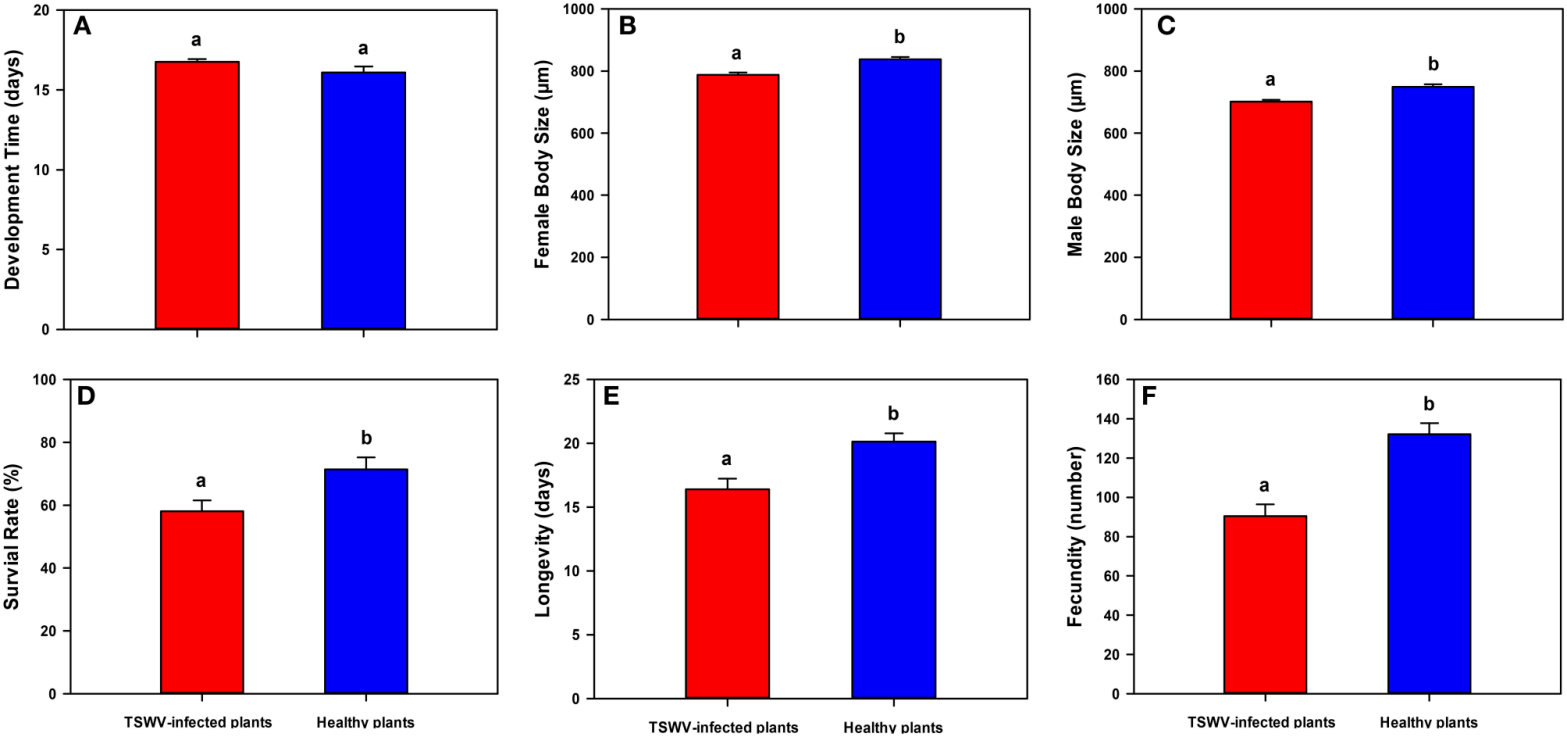
**Life history traits of *B. tabaci* Q on healthy vs. TSWV-infected *D. stramonium*. (A)** Development time (from egg-to-adult) (*n* = 12). **(B)** Female body length (from head to tip of abdomen) (*n* = 50). **(C)** Male body length (from head to tip of abdomen) (*n* = 50). **(D)** Survival (the total number of emerged adult whiteflies/the total number of eggs ^*^ 100) (*n* = 12). **(E)** Longevity of newly emerged adults (*n* = 30). **(F)** Fecundity (the total number of eggs laid per female) (*n* = 30). Values are means ± SE (One-way ANOVA). Within each panel, different letters indicate significant differences between treatments (*P* < 0.05).

Although, TSWV-infected *D. stramonium* did not affect *B. tabaci* Q developmental time [One-way ANOVA, *F*_(1, 22)_ = 2.599, *P* = 0.124; Figure 2A], it indeed decreased female body length [One-way ANOVA, *F*_(1, 98)_ = 20.295, *P* < 0.001; Figure 2B], male body length [One-way ANOVA, *F*_(1, 98)_ = 21.322, *P* < 0.001; Figure 2C], survival rate (egg-to-adult) [One-way ANOVA, *F*_(1, 22)_ = 6.615, *P* = 0.019; Figure 2D], longevity [One-way ANOVA, *F*_(1, 58)_ = 12.570, *P* < 0.001; Figure 2E], and fecundity [One-way ANOVA, *F*_(1, 58)_ = 25.502, *P* < 0.001; Figure 2F].

### *B. tabaci* Q settling and oviposition preferences on TSWV-infected and healthy peppers

**Figure 3:**
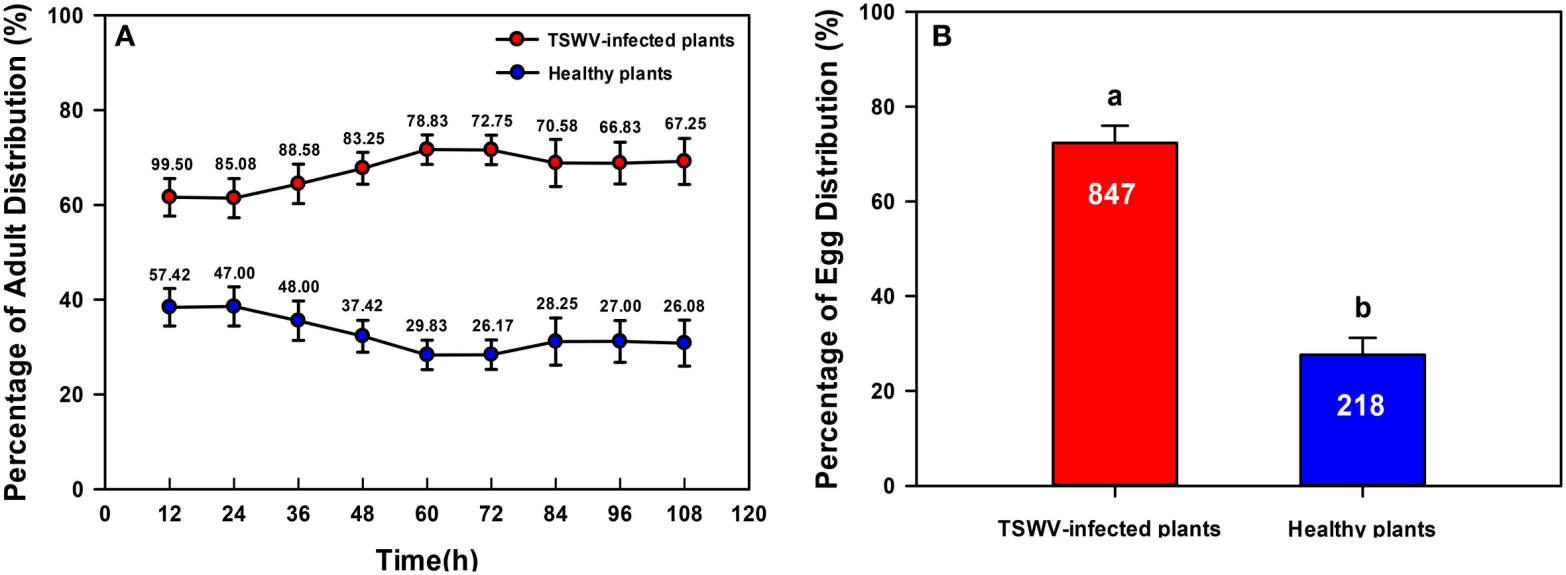
**Settling and oviposition of *B. tabaci* Q on healthy vs. TSWV-infected pepper**. **(A)** Percentage of adults that settled on healthy vs. TSWV-infected plants (*n* = 300, 12 replicates). **(B)** Percentage of eggs distributed on healthy vs. TSWV-infected plants. Values are means ± SE (One-way ANOVA). The number of eggs was shown in the figure. Settling and oviposition were significantly greater on the TSWV-infected plants than on the healthy plants. Within each panel, different letters indicate significant differences between treatments (*P* < 0.05).

Significantly more *B. tabaci* Q adults settled on TSWV-infected plants than on healthy plants [One-way ANOVA, *F*_(1, 22)_ = 52.002, *P* < 0.001; Figure 3A]. Oviposition was greater on TSWV-infected plants than on healthy plants [One-way ANOVA, *F*_(1, 22)_ = 114.406, *P* < 0.001; Figure 3B].

### Volatiles from TYLCV-infected, TSWV-infected, and healthy *D. stramonium*

The most significant different volatiles emitted by *D. stramonium* were o-xylene, α-pinene, phenol, and 2-ethyl-1-hexanol. The quantities of o-xylene, α-pinene, and 2-ethyl-1-hexanol collected differed significantly in virus-infected vs. healthy plants (Table 1). TYLCV-infested plants released significantly more phenol than healthy plants or TSWV-infected plants. None of the other volatiles detected differed between these three groups. TYLCV-infected plants and TSWV-infected plants released significantly more 2-ethyl-1-hexanol than healthy plants. Concentrations of four volatiles in paroline (XiLong, China) were used to test the preference of white flies: o-xylene (1:500,000), α-pinene (1:1,000,000), phenol (1:2,500), and 2-ethyl-1-hexanol (1:2,500; Table 1).

**Table 1:** **Volatiles emitted by virus-infected and healthy *D. stramonium***.

**Compound**	REL. RET. TIME (min)^*^	**CAS#**	**Healthy (Mean ± SE)**	**TYLCV-infected**	**TSWV-infected**	**Test concentration (compound ratio)**
				**(Mean ± SE)**	**(Mean ± SE)**	
o-xylene	1.269	95-47-6	7.20 ± 0.43^a^	2.65 ± 0.40^b^	2.34 ± 0.26^b^	63.52 (1:500,000)
Styrene	1.353	100-42-5	0.38 ± 0.09^a^	0.59 ± 0.09^a^	0.51 ± 0.11^a^	
m-xylene	1.372	108-38-3	1.30 ± 0.30^a^	1.14 ± 0.22^a^	1.40 ± 0.28^a^	
α-pinene	1.604	80-56-8	3.63 ± 0.18^a^	1.30 ± 0.19^b^	1.30 ± 0.10^b^	18.84 (1:1,000,000)
benzaldehyde	1.621	100-52-7	0.65 ± 0.13^a^	0.71 ± 0.06^a^	0.60 ± 0.10^a^	
Phenol	1.818	108-95-2	0.29 ± 0.041^a^	14.42 ± 1.60^b^	0.44 ± 0.10^a^	72.90 (1:2,500)
2-ethyl-1-hexanol	2.01	104-76-7	16.85 ± 1.73^a^	34.88 ± 3.71^b^	38.81 ± 3.46^b^	240.04 (1:2,500)
δ-3-carene	2.103	13466-78-9	0.20 ± 0.11^a^	0.17 ± 0.08^a^	0.16 ± 0.08^a^	
1,4-diethyl-benzene	2.12	105-05-5	0.58 ± 0.34^a^	0.65 ± 0.33^a^	0.52 ± 0.10^a^	
Cymene	2.25	25155-15-1	1.22 ± 0.21^a^	1.46 ± 0.43^a^	1.09 ± 0.26^a^	
Nonanal	2.302	124-19-6	0.89 ± 0.14^a^	1.11 ± 0.06^a^	1.08 ± 0.17^a^	
m-cymene	2.389	535-77-3	1.72 ± 0.73^a^	1.63 ± 0.36^a^	1.53 ± 0.54^a^	
p-cymene	2.534	99-87-6	2.00 ± 0.26^a^	2.15 ± 0.39^a^	2.13 ± 0.71^a^	

* *The retention time of the internal standard, n-octane, was 7.294 min. The four compounds used in the subsequent behavioral assays, including o-xylene, α-pinene, phenol, and 2-ethyl-1-hexanol, were compared with their respective authentic standards (Sigma-Aldrich, USA)*.

*Different letters indicate significant differences (P < 0.05)*.

### Behavioral responses of *B. tabaci* Q to volatiles emitted from *D. stramonium*

**Figure 4:**
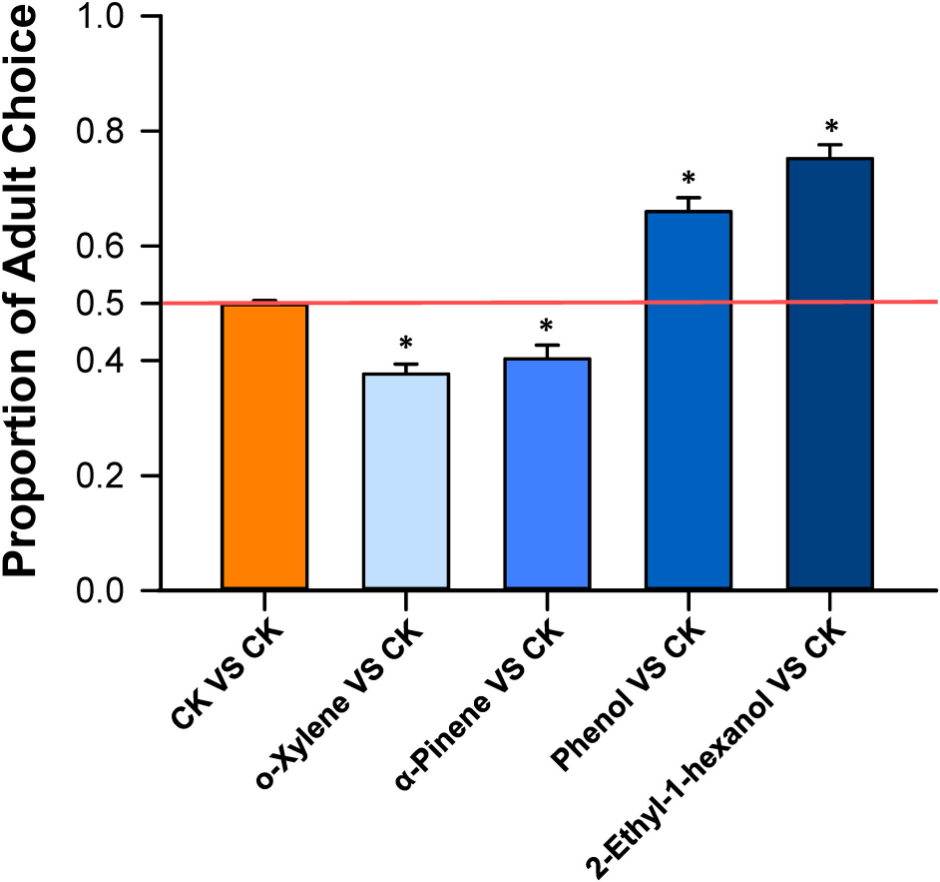
***Bemisia tabaci* Q choice test with volatiles from *D. stramonium*.** Y-tube olfactometer test documented the percentage of *B. tabaci* Q females that selected *D. stramonium* volatiles, including o-xylene, α-pinene, phenol, and 2-ethyl-1-hexanol, over the paroline control, respectively. Values are means ± SE (One-sample *t*-test). Asterisks indicate significant differences (*P* < 0.05).

In dual-choice assays, the proportion of *B. tabaci* Q adults that selected o-xylene or α-pinene was significantly less than the controls (*T*-test, *T* = −7.17, df = 19, *P* < 0.001 for o-xylene; *T*-test, *T* = −4.10, df = 19, *P* < 0.001 for α-pinene), suggesting that adults were repelled by these two chemicals (Figure 4). Healthy plants emitted a greater quantity of these volatiles than virus-infected plants (Table 1). The adults were significantly attracted to phenol (*T*-test, *T* = 6.55, df = 19, *P* < 0.001), and 2-ethyl-1-hexanol (*T*-test, *T* = 10.37, df = 19, *P* < 0.001), respectively (Figure 4). Virus-infected plants, in general, released a greater quantity of these volatiles than the healthy plants (Table 1).

### *B. tabaci* Q free-choice bioassay with semiochemicals

**Figure 5:**
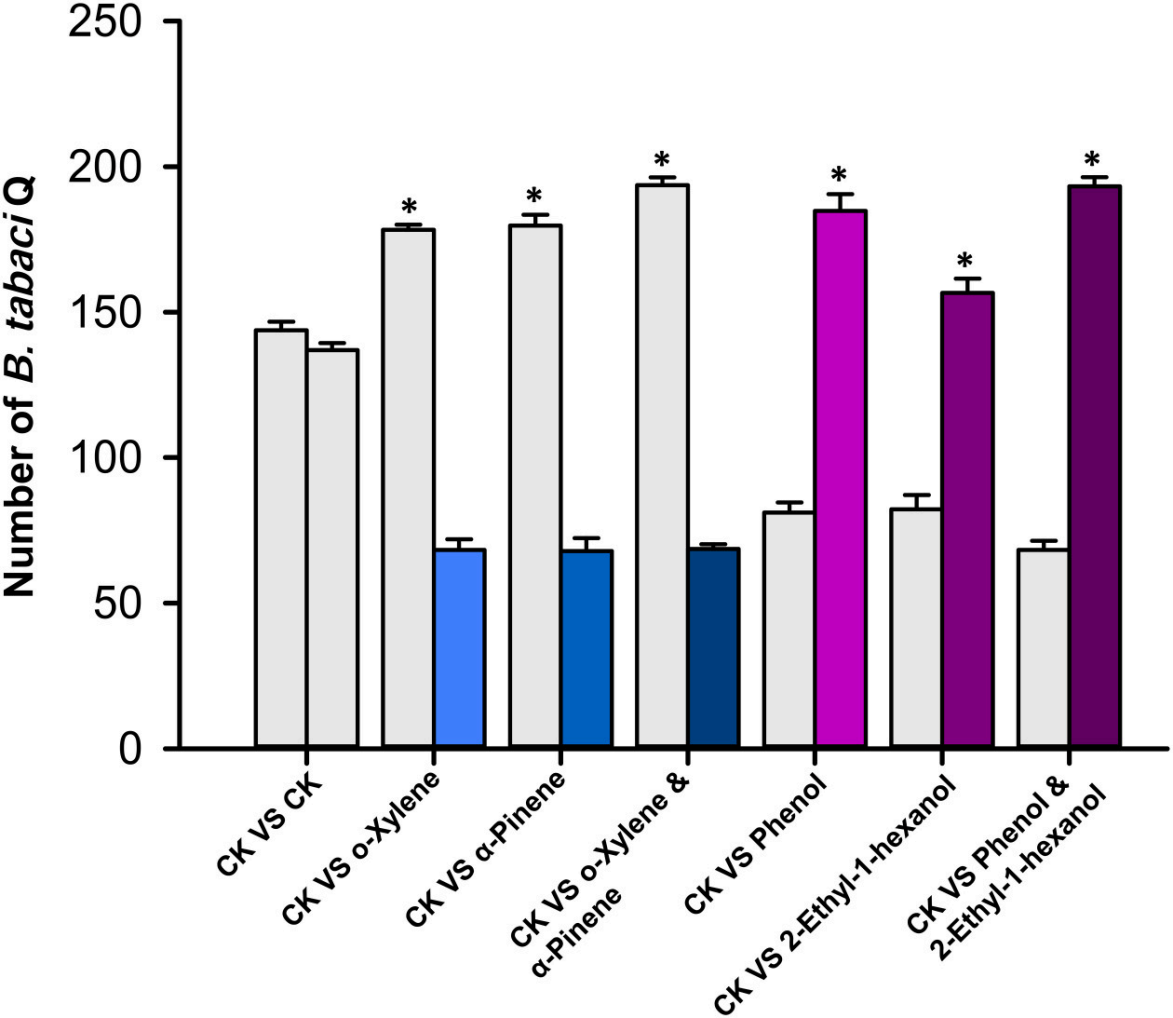
***Bemisia tabaci* Q free-choice bioassay with volatiles from *D. stramonium*.** Number of adults that settled on plants after 20 min. Values are means ± SE (One-way ANOVA). Asterisks indicate significant differences (*P* < 0.05).

To examine the correlation between the four semiochemicals and *B. tabaci* behavior, we assayed host preference using commercially available pure compounds. The selected pure components were administered on paper cards to *D. stramonium* plants (as described above). Two time points (10 and 20 min) were tested in this experiment. The number of recaptured *B. tabaci* on each of the two *D. stramonium* plants did not deviate significantly (Figure 5; the time point selection refers to Bleeker et al., 2009). However, when applied 50 μl of o-xylene or α-pinene solution onto the paper cards, *D. stramonium* became significantly more repellent to the whiteflies compared to the controls (One-way ANOVA, *P* < 0.001; Figure 5). Specifically, *D. stramonium* with o-xylene and α-pinene repelled 97 and 112 whiteflies, respectively, whereas a mixture of both deterred 131 whiteflies (Figure 5). In contrary, the addition of phenol or 2-ethyl-1-hexanol rendered *D. stramonium* significantly more attractive to the whiteflies (One-way ANOVA, *P* < 0.001; Figure 5). Phenol, 2-ethyl-1-hexanol, and their mixture attracted 103, 59, and 119 whiteflies, respectively (Figure 5).

## Discussion

We found that TSWV infection of *D. stramonium* increased the settling and oviposition of the non-vector *B. tabaci* Q on *D. stramonium* even though some of its life history traits were reduced. These results coupled with a previous study (Chen et al., 2013) indicate that virus-infection may, in some cases, cause females to select hosts that do not necessarily maximize their performances. The host selection may depend on the by-products of host infection by the virus. We further tested the hypothesis using peppers, and again found that the non-vector *B. tabaci* Q elected to settle and oviposit on virus-infected pepper. Similarily, *B. tabaci* Q preferred TSWV-infected over healthy peppers, even though TSWV decreased Q performance (Pan et al., 2013). Our results also demonstrated that four volatiles released from TYLCV-infected and TSWV-infected plants (o-xylene, α-pinene, phenol, and 2-ethyl-1-hexanol) can repel or attract *B. tabaci* Q in ways that are consistent with the effects of virus infection on host behavior (Figures 4, 5). In other words, virus-infected plants tended to release greater quantities of volatiles to attract *B. tabaci* Q adults, while healthy plants tended to release greater quantities of volatiles to fend off *B. tabaci* Q adults.

From an evolutionary viewpoint, we expect females to maximize their fitness by laying eggs on plants that maximize offspring's fitness (Gripenberg et al., 2010). However, these expectations may or may not be realized on virus-infected plants. In our previous study, *B. tabaci* Q preferred to settle and oviposit on TYLCV-infected rather than on healthy *D. stramonium* and had greater fitness when feeding on TYLCV-infected *D. stramonium*, suggesting an indirect mutualism between *B. tabaci* Q and TYLCV (Chen et al., 2013). Similar phenomenon has been documented in other insect-virus systems (Montllor and Gildow, 1986; Castle and Berger, 1993; Jiménez-Martínez et al., 2004a; Srinivasan et al., 2006; Ngumbi et al., 2007). In our case, *B. tabaci* Q preferred virus-infected *D. stramonium*, however, the overall performance of whiteflies on TSWV-infected plants was reduced. Although, we did not examine other traits, including female cost for oviposition or offspring protection from natural enemies, which also contribute to insect fitness, our results demonstrate a deviation from the mother-knows-best hypothesis. Under the ecological and evolutionary context, we speculate that the apparent “loss” for *B. tabaci*, a non-vector of TSWV, represents a “gain” for TSWV. By reducing the fitness/performance of *B. tabaci*, the insect vector for many other plant viruses, the transmission of competing pathogens will be compromised, which means a limited or no sharing of host plants for TSWV.

Mauck et al. (2012) suggested that vectors prefer to settle on plants infected with persistently transmitted (PT) viruses, while non-persistently transmitted (NPT) viruses generally have no effect on vector settling and feeding or cause infected plants to be less attractive than healthy plants. Mauck and his colleagues believe that PT viruses typically enhance host plant quality for vector herbivores, resulting in enhanced vector herbivore fitness. NPT viruses, in contrast, often decrease or have no effect on host plant quality (Mauck et al., 2012). For example, pepper plants infected with TSWV increased the survival and oviposition of the spider mite *Tetranychus urticae* (Belliure et al., 2010). The growth of *Spodoptera exigua* caterpillars was accelerated on tomato plants infected with *Tobacco mosaic virus* (Thaler et al., 2010). Our previous study showed the enhanced *B. tabaci* performance on TYLCV-infected *D. stramonium* (Chen et al., 2013). In this study, however, we demonstrated that quality of the virus-infected plants might not be the deciding factor for *B. tabaci* to choose the host plants.

To test whether our results applied to other hosts, we determined whether TSWV infection affected *B. tabaci* Q settling (Figure 3A) and oviposition (Figure 3B) on pepper. The results with pepper were the same as those with *D. stramonium* in terms of settling and oviposition preferences. Consistent with our finding, several previous studies indicated that *Cucumber mosaic virus* (CMV) significantly decreases host quality (the aphid *Myzus persicae* and *Aphis gossypii* performed poorly on infected host plants) but enhances the attractiveness of infected host plants to aphids (Mauck et al., 2010). We suggest that regardless of how virus infection influences insect fitness, both vector and non-vector insects may prefer to settle and oviposit on virus-infected plants rather than on healthy plants, i.e., both vectors and non-vectors may be “manipulated” by viruses. Although, the ecological relevance of such manipulation of non-vectors is unknown, we speculate that these non-vector insects might be victimized by the co-evolution between viruses and their vectors due to the shared chemical signatures emitted by the virus-infect plants.

Our results also suggest that the preference of *B. tabaci* Q for virus-infected plants may be mediated by virus-induced changes in plant volatile emissions. Virus-induced effects on plant odor cues are the induction of specific chemical emissions by plant pathogens (Anderson and May, 1991; Jiménez-Martínez et al., 2004b). For example, enhanced preference of sandflies to hamsters who infected with *Leishmania* was attributed to changes in host-derived odor cues (O'Shea et al., 2002), and it is considered that volatiles also might explain a study result that the children from western Kenyan harboring the gametocytes (the stage transmissible to mosquitoes) of the *Plasmodium falciparum* attracted strongly more *Anopheles gambiae* than uninfected children or those harboring the asexual (non-infective) stage of the parasite (Lacroix et al., 2005). The pathogen causing Dutch elm disease has been revealed to up-regulate volatiles that attract vectors *Hylurgopinus rufipes* to infected host trees (McLeod et al., 2005). van Molken and his colleagues found that virus infection reduces the attractiveness of host plants for a non-vector insect (van Molken et al., 2012), suggesting the ecological benefits of virus infection for host plants by inducing insect repellence. Our results, however, showed that virus infection attracted a non-vector insect to the host plant. Additional experiments are warranted to resolve this discrepancy.

In summary, this study provides empirical evidences showing that virus-infection increases insect settling and oviposition but reduces insect performance; the long-term evolutionary consequences of these effects are unclear. Specifically, the preference for virus-infected plants in *B. tabaci* is correlated with an increase in phenol and 2-ethyl-1-hexanol and a decrease in o-xylene, α-pinene, within the volatile profile emitted by *D. stramonium* plants. As a non-vector of TSWV, however, insect performance seems to be impaired since adult body size, longevity, survival, and fecundity diminished in TSWV infected *D stramonium* plants.

Nevertheless, other factors may affect host selection as well. Trichome plays an important role in the host selection process, ovipositional behavior, and larval performance (Shanower et al., 1999; Mayhew, 2001; Handley et al., 2005). Trichomes and their exudates can reduce the efficiency of natural enemies and thus may have negative or positive influences on insect herbivory insect populations (Blaney and Simmonds, 1990). Besides there are four major groups of defensive compounds in plants that impact insect performance (Schoonhoven et al., 1998), including phenolics (e.g., polyacetates, tannins, and lignins), terpenoids (e.g., cardenolides and terpenes), cyanogenic glycosides and glucosinolates, and nitrogen-containing compounds (e.g., non-protein amino acids and alkaloids). These compounds can have both direct and indirect influences on the performance of herbivorous insects (Awmack and Leather, 2002). TSWV infection may induce changes in trichome traits or the release of defensive compounds in host plants, which warrants further investigation.

## Materials and methods

### *Bemisia tabaci* Q laboratory population

The *B. tabaci* Q used in this study was originally collected from a poinsettia plant in Beijing, China, in 2009 and it has been maintained on poinsettia (*Euphorbia pulcherrima* Wild. ex Klotz.) in insect-proof cages at 26 ± 2°C with a 12/12 h light/dark photoperiod. The identity of whitefly cryptic species was confirmed by cleavage amplified polymorphic sequence (CAPS) and mitochondrial cytochrome oxidase I genes (*mtCOI*; Chu et al., 2010).

### Plant cultures and TSWV inoculation

*D. stramonium* L. and pepper (*Capsicum annuum* L., cv Zhongjiao 6) plants were grown as described previously (Chen et al., 2013). TSWV was maintained on *D. stramonium*. The virus inoculum on *D. stramonium* and pepper plants were done as described by Pan et al. (2013). Infection was then confirmed for inoculated and non-inoculated control plants by ELISA as described by Chen et al. (2013). When these plants grew to the seven true-leaf stage, they were used in the experiments.

### *Bemisia tabaci* Q settling and oviposition on healthy vs. TSWV-infected plants

Experiments concerning whitefly settling and oviposition preference were performed as described by Chen et al. (2013). There were 12 replicate cages for each combination of plant species and virus status (±infected), with insects and plants drawn from the same pool. These replicates were distributed over time and independent. The replicate cages were simultaneously arranged with each side exposed to natural lighting in a greenhouse at 26 ± 2°C. Individual plants of healthy and TSWV-infected *D. stramonium* or pepper were arranged in opposite corners of the cage. Approximately 300 adult *B. tabaci* Q were released in the center–bottom of each cage.

### *Bemisia tabaci* Q life history traits on healthy vs. TSWV-infected *D. stramonium*

Experiments concerning life history traits of whitefly were performed as described by Chen et al. (2013). The development time, female body length, male body length, survival, longevity of newly emerged adults and fecundity were evaluated in these experiments.

### Extraction and analysis of volatiles emitted by TYLCV-infected, TSWV-infected, or healthy *D. stramonium*

Experiments concerning volatiles were extracted and analyzed as described by Wei et al. (2007). Volatiles emitted from uninfested plants and plants that have been infested by TYLCV or TSWV were collected using a headspace collection system in a climate room equipped with high-intensity sodium-halide lights (temperature 24 ± 2°C; RH 60–70%). The soil containing the roots of each plant was carefully wrapped in aluminum foil, and the plant was placed individually in a 2.5-L glass jar with a gas inlet and a gas outlet. Each of the three treatments was represented by five replicate plants. Before air (78% nitrogen, 21% oxygen, and 1% carbon dioxide) was pumped into each glass jar through the inlet, it was purified through a gas purge system. A glass tube filled with 100 mg of PoraPak Q (80/100-mesh; Waters, USA) was used to trap plant volatiles at the outlet. After 4 h collection under continuous light, volatiles were eluted from the PoraPak Q with 800 μL of high-performance liquid chromatography (HPLC)-grade methylene chloride (Tedia Company, Fairfield, Ohio, USA), and samples received 10 μL of an internal standard (20 ng/μL of n-octane) used for quantifying relative compound amounts. Then a 1-μL sample of the solution was subjected to gas chromatography–mass spectrometry (GC-2010 Shimadzu, Japan). The system was equipped with Agilent Technologies capillary column DB-5MS (30 m × 0.25 mm ID × 0.25 μm film thickness).

For analyses using the Agilent Technologies capillary column, the initial oven temperature was kept at 40°C for 4 min and then increased to 180°C at a programmed rate of 5°C min^−1^, followed by a rate of 10°C min^−1^ to 230°C. On a DB-5MS column (30 m × 0.25 mm ID × 0.25 μm film thickness), the GC oven temperature was kept at 40°C for 4 min and then increased to 200°C at a rate of 5°C min^−1^, followed by a rate at 20°C min^−1^ to 280°C. The inlet was operated under the splitless injection mode, and the injector temperature was maintained at 250°C with a constant flow rate of 1.0 mL min^−1^. The GC-MS electron impact source was operated in the scan mode with the MS source temperature at 230°C and the MS Quad at 150°C. Compounds were identified by comparison of retention time and mass spectra (NIST database and synthetic standards). The following volatiles were detected: o-xylene, α-pinene, phenol, and 2-ethyl-1-hexanol. The peak area of the volatile expressed as a proportion of the peak area of the internal standard was used for quantification.

### *Bemisia tabaci* Q choice tests with volatiles from *D. stramonium*

The preferences of *B. tabaci* Q for the major volatiles released from *D. stramonium* (o-xylene, α-pinene, phenol, and 2-ethyl-1-hexanol; see previous section) were investigated using a Y-tube olfactometer. The solvent paraffin oil was included as a vehicle control. The working concentrations of o-xylene (1:500,000), α-pinene (1:1,000,000), phenol (1:2,500), and 2-ethyl-1-hexanol (1:2,500) were five to eight-fold equivalent in *D. stramonium* (Table 1). Two streams of purified air (filtered through activated charcoal) were passed through two glass containers (one contained 1 ml solution of the test volatile in paroline and the other contained only 1 ml paroline as a control) into the olfactometer arms at 400 ml min^−1^.

The experiment started with the placement of thirty *B. tabaci* Q females at the base of the Y-tube. Each test was observed for 30 min, and a choice for one of the two odor sources was recorded when the whitefly reached the end of one arm and stayed there for at least 15 s after 30 min. After finishing each test, odor sources were interchanged to avoid any influence of unforeseen asymmetries in the set-up. There were five treatment comparisons: paroline (CK) vs. CK, CK vs. o-xylene, CK vs. α-pinene, CK vs. phenol, and CK vs. 2-ethyl-1-hexanol. This experiment was performed 20 times per comparison, 10 times each day (between 9:00 and 15:00).

### *Bemisia tabaci* Q free-choice bioassays with volatiles from *D. stramonium*

Experiments concerning *B. tabaci* Q free-choice bioassays were tested as described by Bleeker et al. (2009) with minor modifications. For bioassays with volatiles (mix or single compounds), two potted tomato plants were placed in a screen cage setup at a distance of 50 cm from each other. Three hundred whiteflies were released in the middle and recorded after 10 and 20 min. To test the effect of selected headspace components on repellence and attraction, 50 μl solution of synthetic standards (Sigma-Aldrich, USA) (the concentration of solution refer to test concentration in Table 1) were applied to 5 filter paper discs (Whatman; 25-mm diameter). The treated (add compound) and controlled (add 50 μl solvent paroline) discs were attached respective to two plants with metal wire. The position of the treated tomato was randomized. Five minutes after placing the volatiles on the plant, whiteflies were released. For each component, eight replications were carried out. Prior to each assay with volatiles, the experiment was done with the two plants in a controlled setup, in the same position to allow comparison in the same background. For each volatile compound tested, a new set of plants was used.

### Data analysis

Repeated-measures ANOVAs were used to compare the whitefly settling preference on healthy vs. TSWV-infected plants (*D. stramonium* and pepper). The assumption of sphericity is confirmed by Mauchly's Test. One-way ANOVAs were used to compare whitefly oviposition preference on healthy vs. TSWV-infected plants (*D. stramonium* and pepper); to compare the life history parameters on the healthy vs. TSWV-infected *D. stramonium* plants; to compare the four standards in different *D. stramonium*; and to compare the result of free-choice bioassays with volatiles. One-sample *t*-tests were used to compare the proportion of whitefly number in the treatment side to the expect proportion of 0.5. SPSS version 19.0 (SPSS Inc., Chicago, IL, USA) was used for all statistical analyses. All proportional data were arcsine square root transformed before analyses.

## Author contributions

GC contributed to the chemical ecological laboratory work, performed the major part of entomological manipulations, participated in data analysis, participated in the conception and design of the study and drafted the manuscript; QS and XS carried out ecological manipulations; XL, ZP, and HZ contributed to viral and microbiological manipulations; WX and BX contributed to insect and plant husbandry and data collection; SW and QW carried out the statistical analyses; YZ and XZ contributed to the conception and design of the study, coordinated the study and edited the manuscript. All authors gave final approval for publication.

## Funding

This work was supported by the State Key Program of National Natural Science Foundation of China (31420103919), the 973 Program (2013CB127602), the National Natural Science Foundation of China (31572014), China Agriculture Research System (CARS-26-10), Beijing Training Project For The Leading Talents in S and T (LJRC201412), Graduate Research and Innovation Project in Hunan Province (CX2015B248) and the Beijing Key Laboratory for Pest Control and Sustainable Cultivation of Vegetables. The granting agencies had no role in study design, data collection and analysis, decision to publish, or preparation of the manuscript.

### Conflict of interest statement

The authors declare that the research was conducted in the absence of any commercial or financial relationships that could be construed as a potential conflict of interest.
